# The influence of ultrasonic shot peening on the microstructure and fatigue behavior of TC17 alloy

**DOI:** 10.1038/s41598-025-89959-1

**Published:** 2025-04-19

**Authors:** Jin Cai, Hao Wu, Yongyi Tian, Xiaoxin Zhang

**Affiliations:** College of Aerospace Engineering, Shenyang Aerospace University, Shenyang 110136, China

**Keywords:** Surface strengthening, Ultrasonic shot peening, Titanium alloy, Uniformity, Quantitative characterization, Strain gradient, Materials science, Engineering, Aerospace engineering

## Abstract

This research presents a quantitative analysis of TC17 alloy subjected to ultrasonic shot peening (USP) treatment. The effects of USP treatment under different Almen intensities on surface roughness, microstructure, plastic strain range, microhardness, residual stress, fatigue behavior and strain gradient of TC17 alloy were analyzed. Results show that increasing Almen intensity from 0.15 mmA to 0.25 mmA leads to a 27% increase in surface roughness, a 12% increase in surface compressive residual stress value, a 29% increase in the depth of compressive residual stress layer, and a 6.7% increase in the maximum value of compressive residual stress. Microstructural analysis reveals material stacking, pores, and microcracks on the specimen surface and grain refinement characteristics in the surface and subsurface layers. Strain gradient analysis shows that the deformation layer depth is uniformly distributed. An increase in Almen intensity leads to a higher degree of plastic deformation and work hardening, and the increase in compressive residual stress layer depth counteracts early fatigue failure caused by high roughness.

## Introduction

Due to their ability to retain high strength at an elevated temperature over the operating life, TC17 alloys are extensively used in key components of aero-engines such as fan disks and compressor disks^1,2^. These components are subjected to extreme operating conditions, which frequently result in the failure of structural components, thereby reducing the service life and reliability of aero-engines. Among these, fatigue failure accounts for over 80% of total failures in aero-engine components^3^. And the main factors affecting the fatigue life of metal materials are microstructure, residual stress, and roughness^4–6^. Ultrasonic shot peening (USP) emerges as a sophisticated surface treatment technique that has garnered increasing attention due to its ability to induce large-depth compressive residual stress onto the surface of metallic components. The compressive residual stress generated by USP has been found to effectively improve the fatigue properties of titanium alloys by refining the surface grains, delaying crack initiation, and slowing down crack propagation rates^7–12^. Concurrently, the surface roughness of the ultrasonic impact treated specimen is relatively low^13–15^.

French Aerospace Institute^16^ proposed that the uniformity of the strain field and work hardening introduced by surface strengthening requires quantitative characterization to accurately assess the change of surface strengthening to the gradient of the material’s hardened layer. MTU Aero Engines AG^17^ proposed that the USP process to produce uniform plastic deformation and consequently achieve a uniform distribution of residual stress and surface macro and micromorphology. USP treatment requires optimizing various process parameters to achieve quantitative control of plastic strain layer depth, surface roughness, stress field, and microstructure uniformity^18^.

Zhang^19^ used USP to treat selective laser melting TC4 titanium alloy. The author found that the microstructure and microstrain of surface can be improved by adjusting the processing time. Tan’s research^20^ on the effects of USP with different shot diameters on the surface in TC17 alloy revealed that larger shot diameters lead to an increase in surface roughness, plastic deformation and a deeper grain refinement layer. Wang^21^ analyzed the effect of different USP process parameters on the residual stress of titanium alloys. The author obtained the effectiveness of adjusting USP parameters on the uniform distribution of the stress field. Xu^22^ studied the effect of USP on the surface roughness of titanium alloys. USP can achieve a surface with low roughness and high quality. Concurrently, Cai^23^ explored the uniformity of the roughness distribution of specimens with different Almen intensities. An increase in the Almen intensity reduced the dispersion of the roughness value distribution. Most of the previous studies focused on the surface topography and surface distribution state. However, they did not quantitatively characterize the strain gradient and uniformity of the microstructure gradient. The objective of this paper is to investigate the impact of USP treatment on the surface roughness, microstructure, plastic strain range, microhardness, residual stress, and fatigue behavior of the TC17 alloy with a strain gradient under 0.15 mmA and 0.25 mmA Almen intensities. Furthermore, this study provides a quantitative characterization of the USP treatment method.

## Materials and methods

**Fig. 1:**
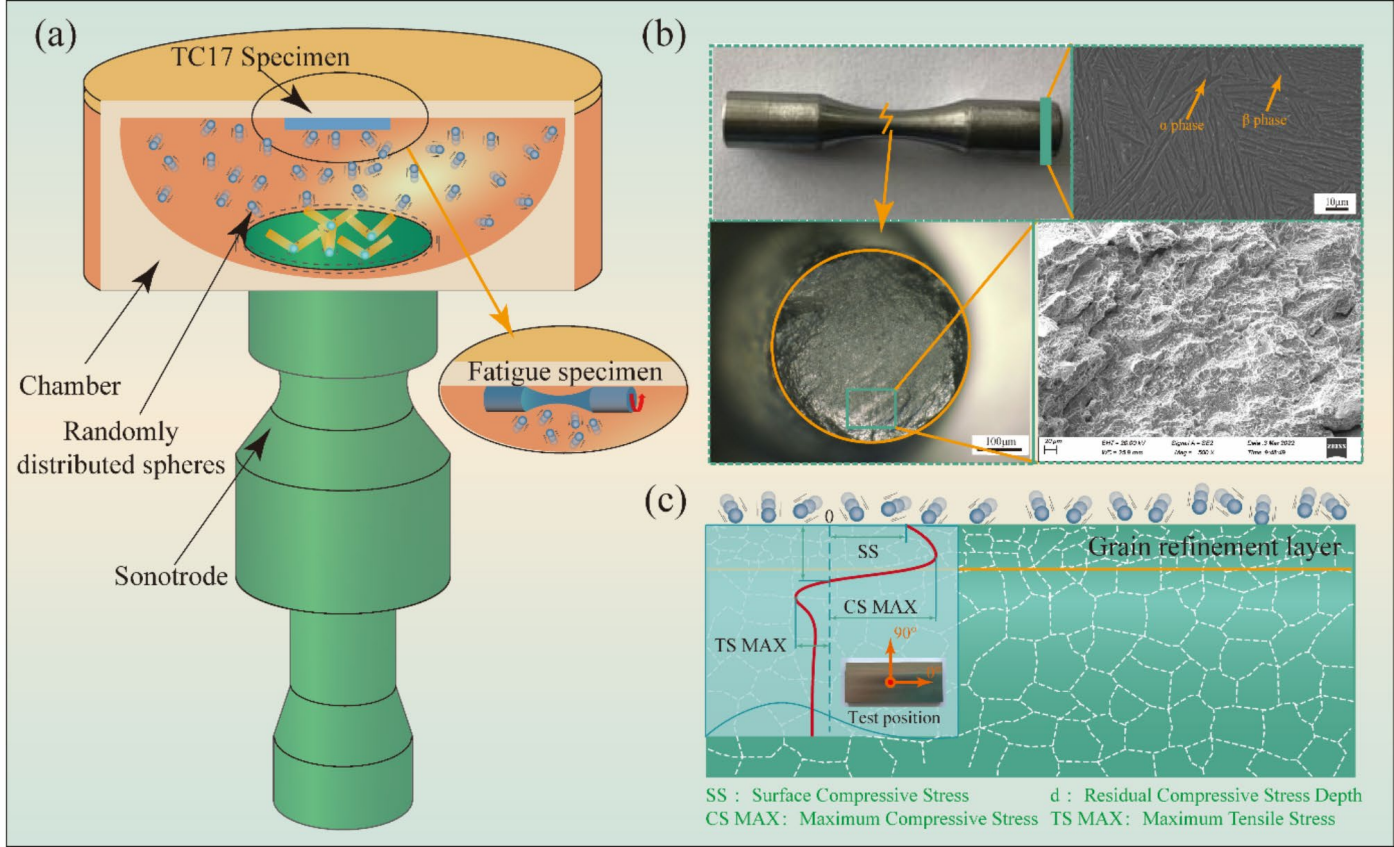
Schematic diagram of USP of TC17 alloy: (**a**) principle of USP device; (**b**) fatigue analysis; and (**c**) result of homogeneity characterization.

The experimental material used in this study was the β-forged TC17 alloy, with a chemical composition listed in Table 1. The substrate microstructure typically exhibited a basket structure, as illustrated in Fig. 1b. The specimens were subjected to annealing at 360 ℃ for 30 min and at 550 ± 5 ℃ for 3–4 h, followed by air cooling. The β-forged TC17 alloy consisted of a needle-like α phase and a coarse β phase, with the α phase interlacing with a small aspect ratio to form a typical basket weave structure. The schematic diagram of the USP treatment is presented in Fig. 1. USP was conducted on TC17 alloy using different Almen intensities, which was defined as the arc height of the Almen test piece used for calibration, and the SAE AMS 2580 A standard document can be consulted for reference. The experiment utilized 2.5 mm diameter zirconia balls, totaling 1000 projectiles. These zirconia ceramic balls boasted a density of 6.00 g/cm³ and a microhardness of ≥ 1250 HV. The sonotrode operated at a frequency of 20 kHz with an amplitude of 60 μm. The treatment durations were 480 s and 960 s, corresponding to Almen intensities of 0.15 mmA and 0.25 mmA, respectively. The principle of USP device is presented in Fig. 1a.

**Table 1:** The chemistry component of TC17 alloy.

Element	Cr	Al	Ti	Zr	Mo	Sn	Fe
Weight(%)	3.72	4.85	83.15	1.72	3.52	2.24	0.2

Surface roughness was measured using a MarSurf 300 C portable roughness measuring instrument at random positions on the treated surface. The mean value, within an assessment length of 4.0 mm, of the maximum peak to valley height (${R_t}$) and the mean spacing of adjacent local peaks (${D_P}$) were used to estimate the geometrical notch stress concentration factor (${K_t}$) by the surface dimples according to the following expression proposed by^24,25^:

1 $${K_t}=1+4{\left( {\frac{{{R_t}}}{{{D_P}}}} \right)^{1.3}}$$

The surface morphology was observed using a Hitachi S-4300 cold field emission scanning electron microscope, while the cross-sectional structure was observed using an Olympus PM3 metallographic microscope. The cross-sectional grain orientation of TC17 after USP alloy was determined using Oxford NordlysNan electron backscatter diffraction. Employing a microhardness tester (HXS-1000 A), thorough measurements of the microhardness distribution were performed at various depths with an interval of 0.03 mm. A load of 25 gf was applied, with a maintenance duration of 10 s. The surface-treated specimens were investigated using an X-ray diffraction (XRD) machine (LXRD, Proto, Canada). The XRD machine requires a voltage of 30 kV and a current of 6.7 mA. The surface of the specimen was scanned with Cu-Kα radiation at a speed of 5° per minute in the range of 30° to 100°, and the measurement range was about a 2 mm diameter spot. The residual stress of the surface was measured using the sin2φ method. The test position is at the center of the specimen, with testing orientations aligned along the 0° and 90° axes of the specimen (Fig. 1c). The residual stress is tested at the surface, and at 5 μm, 10 μm, 20 μm, 30 μm, 40 μm, 50 μm, 70 μm, 90 μm, 120 μm, 150 μm, 200 μm, and 230 μm positions along the length of the specimen, respectively. To carry out the residual stress test in the depth direction, a corrosion method was employed, utilizing a corrosion solution composed of HF (30 mL) + HNO_3_ (10 mL) + HCl (10 mL) + H_2_O (50 mL). Rotational bending fatigue tests were conducted using a QBWP-1000 rotary complete fatigue testing machine. The fatigue test was performed on a fatigue test bar at room temperature, following the GB/T 437–2008 standard. The test was conducted at a frequency of 80 Hz and a stress ratio of *R* = -1. The fracture analysis of the fatigue specimens was observed using a Hitachi Su3500 scanning electron microscope.

## Results and discussion

### Surface roughness and microstructure

**Fig. 2:**
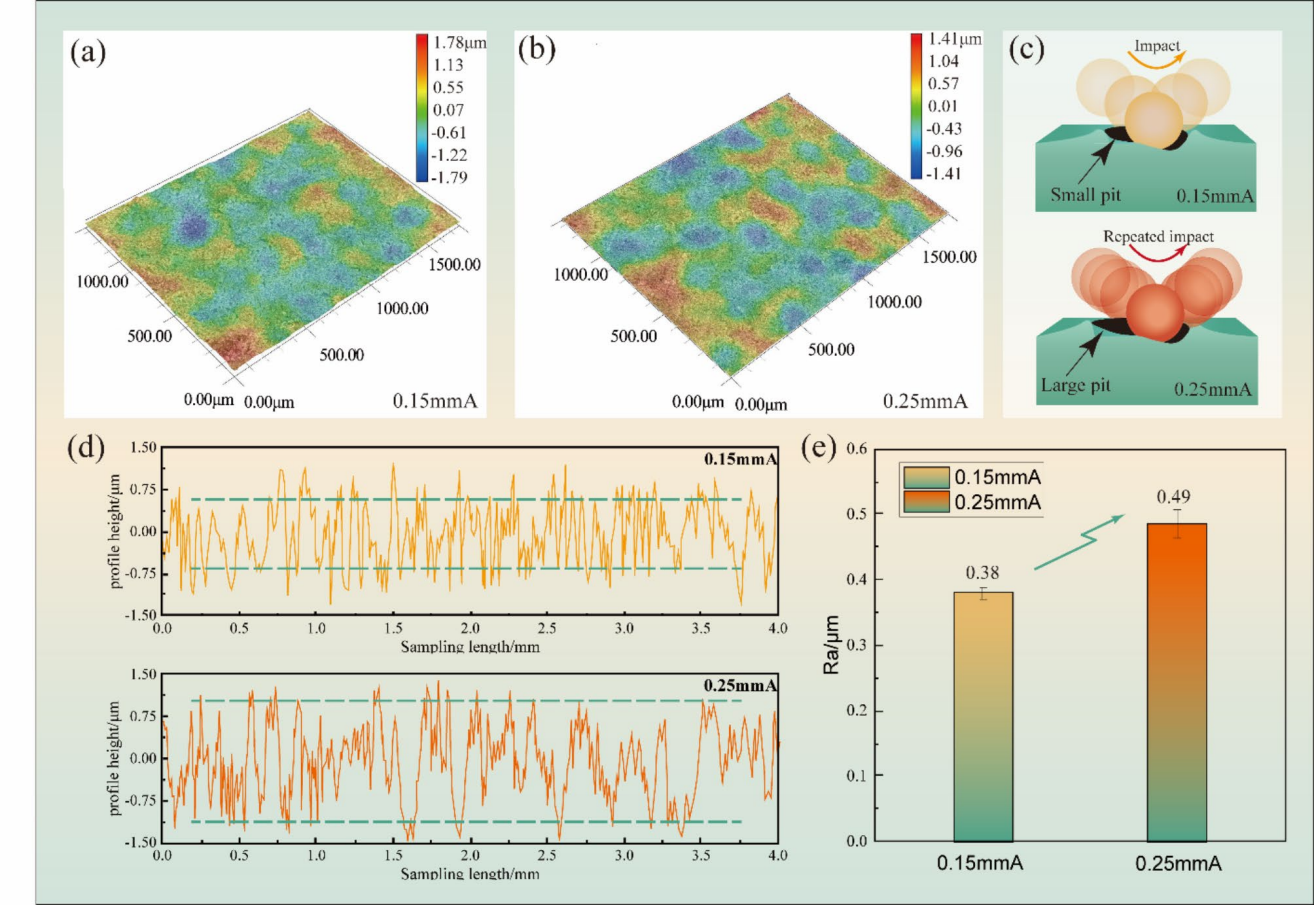
Surface morphology and roughness of TC17 alloy with different Almen intensities: (**a**,**b**) surface morphology; (**c**) the pit after different impact; (**d**) 2D roughness profiles; and (**e**) surface roughness of TC17 alloy after USP treatment.

The surface roughness profile of TC17 alloy after USP treatment with different Almen intensities is depicted in Fig. 2a, b, showing the presence of locally appearing pits (blue) and protrusions (red). Figure 2c shows the pit after different impact. Within a certain range, an increase in peening duration results in an increase in the number of pits and exacerbation of surface protrusions and pits, ultimately leading to an increase in surface roughness of titanium alloy. Within a certain range, an increase in the peening duration led to an increase in the number of pits and an increase in the surface roughness of the titanium alloy, as observed in the study by Lihua Zhu^26^. Compared to the low Almen intensity, a higher number of pits were observed on the surface of the high Almen intensity specimen, with an increase in the height of the bumps around the pits and the depth of the pits. During USP, the high-speed projectiles randomly impacted the TC17 alloy, causing plastic deformation by extrusion and resulting in the formation of protrusions and depressions on the material surface. Similar to the study by Qi Zhang^27^, an increase in the Almen intensity led to an increase in the average roughness of the USP-treated specimens. The size and number of pits on the surface of TC17 alloy also increased with the Almen intensity, and the average and discrete degrees of surface roughness of the alloy also increased, as shown in Fig. 2d, e. Surface roughness and its notch stress concentration factor are listed in Table 2. It is observed that, despite the different roughness characteristics for specimens with different Almen intensities, the notch effects of both surface reliefs are similar. There is an analogy with the result by Mordyuk^28^. On the contrary, the ${K_t}$ value for 0.15 mmA is slightly higher than that for 0.25 mmA. Thus, this similarity allows using the fatigue SN curves data confidently and puts the other possible reasons for the fatigue enhancement in the forefront.

**Table 2:** Surface roughness and its notch stress concentration factor.

Almen intensities	${R_a}$ (µm)	${R_t}$ (µm)	${D_P}$ (µm)	${K_t}$
0.15 mmA	0.38	2.63	77	1.05
0.25 mmA	0.49	2.81	105	1.04

**Fig. 3:**
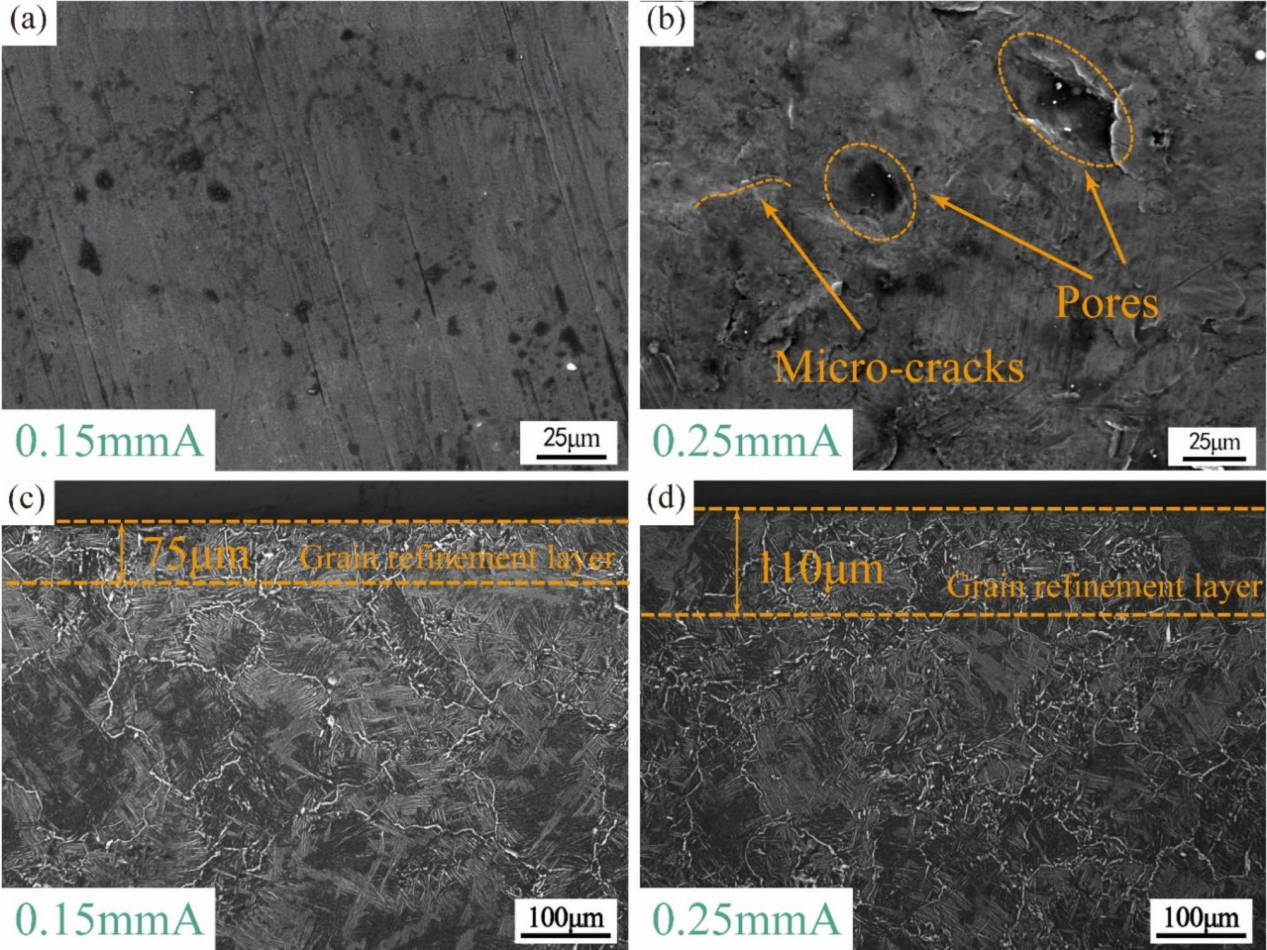
Surface morphology and cross-sectional microstructure distribution of TC17 alloy with different Almen intensities: (**a**,**b**) surface morphology; (**c**,**d**) cross-sectional microstructure distribution.

The surface morphology and cross-sectional microstructure distribution of TC17 alloy with different Almen intensities are presented in Fig. 3. Machining marks were observed on the specimen surface, which were weaker than the surface state of the original structural parts. Additionally, no new local deformation or cracks were introduced on the surface under 0.15 mmA. However, for the 0.25 mmA specimen, the size and depth of the surface crater were relatively large, and the adjacent area limited the extension of the plastic deformation layer, resulting in the appearance of phenomena such as material stacking, pores, and micro-cracks in the local area, as shown in Fig. 3b. Moreover, obvious hole features were present in the local area, which could easily cause local stress concentration and crack initiation points. The grain size distribution in the near-surface area of the specimen section was noticeable after USP treatment, while the depth of the deformed layer was uniform. Similar to the study by Zhang Q^29^, USP treatment led to surface plastic deformation and grain refinement. The grain refinement layer could be observed after USP treatment with Almen intensities of 0.15 mmA and 0.25 mmA, with a depth of approximately 75 μm and 110 μm, respectively, while the grain size gradient increased along the depth direction. Compared to the low Almen intensity, the grain gradient distribution of the high Almen intensity specimen extended along the depth direction, and the grain deformation layer caused by USP treatment was more widely distributed. This observation aligns with other studies that have documented the phenomenon of grain refinement following USP treatment across a range of materials^30,31^.

### Fatigue analysis

**Fig. 4:**
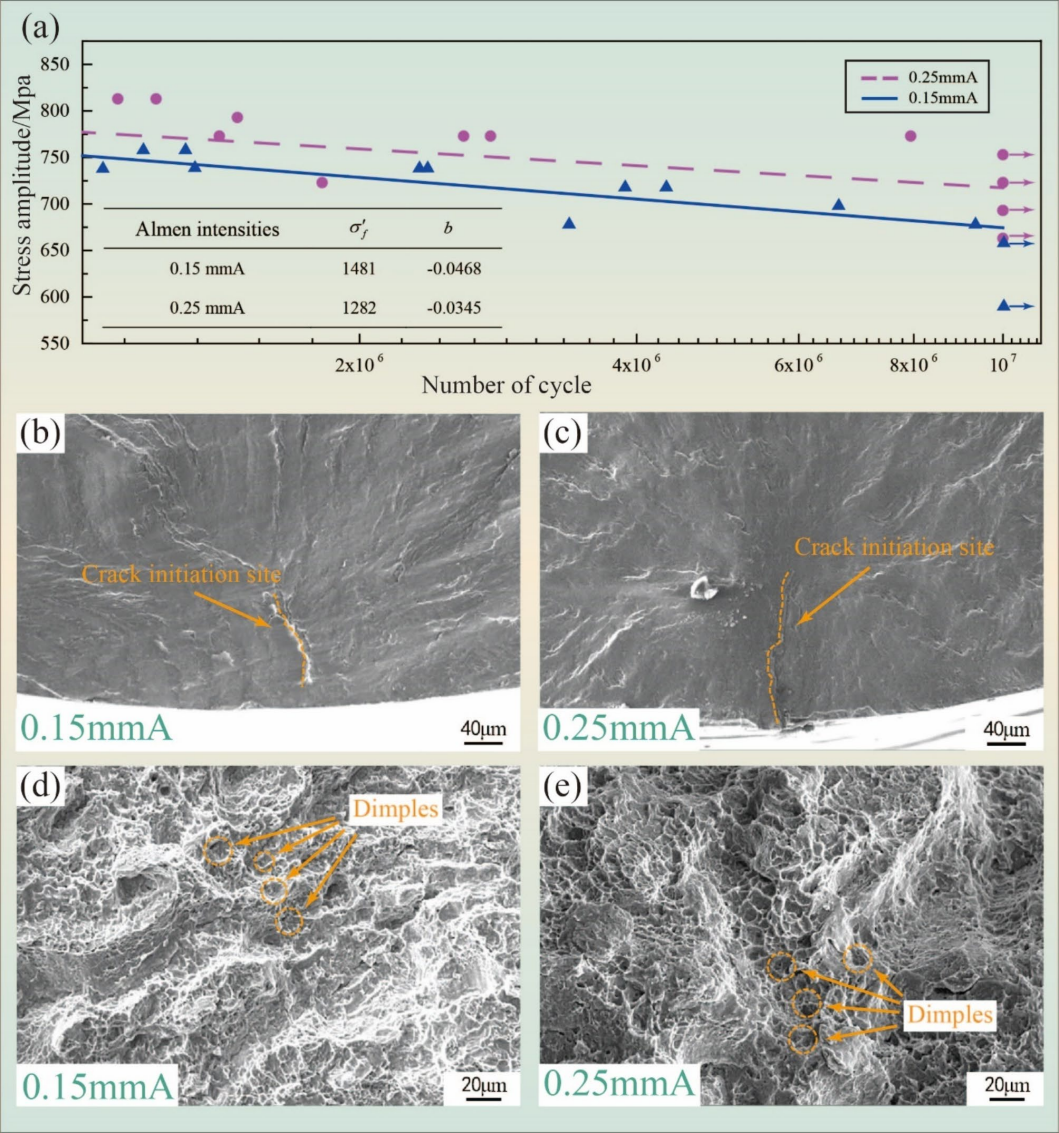
High cycle fatigue SN curves and fracture morphology of TC17 alloy: (**a**) high cycle fatigue SN curves of TC17 alloy; (**b**,**c**) fatigue source area with different Almen intensities; (**d**,**e**) final fracture area with different Almen intensities.

Figure 4 shows the high cycle fatigue SN curves and fracture morphology of TC17 alloy. The high cycle fatigue SN curves of the TC17 alloy under different Almen intensities are presented in Fig. 4a. Results were approximated by the Basquin function (Eq. (2)) with use of least square method:

2 $${\sigma _a}={\sigma ^{\prime}_f} \times {({N_f})^b}$$

Where ${\sigma _a}$ is the stress amplitude, ${\sigma ^{\prime}_f}$ is the coefficient of fatigue toughness, ${N_f}$ is the number of cycles to failure and *b* is the exponent of fatigue life curve. Coefficients of regression curves for both types of specimen are in Fig. 4a.

Based on the fatigue test results and regression curves trend, it is evident that the curves begin to converge around *N* = 6 × 10^5^ cycles. For *N* > 6 × 10^5^ (high cycle region) it is possible to affirm that the fatigue strength will be lower for the 0.15mmA specimens than for the 0.25mmA specimens. It can be seen that fatigue strength of experimental specimens after 0.25mmA treatment increases in the high cycle region and the fatigue strength improvement gets higher with the number of cycles. This is also obvious on the values of coefficient of fatigue toughness ${\sigma ^{\prime}_f}$, which is the stress value in which the regression curve reaches the y-axis. The value ${\sigma ^{\prime}_f}$ for 0.15mmA is equal to 1481 MPa, higher than the value of the 0.25mmA, which is 1282 MPa. This observation is supported by the research conclusion of Wu^32^. Additionally, it is observable that the fatigue limit of 0.25 mmA is approximately 713 MPa, roughly 3.5% higher than that of 0.15 mmA.

Figure 4b, c depict the fracture morphologies of the fatigue source region under different Almen intensities. The fatigue source area is affected by wear and oxidation in high cycle fatigue, resulting in darker colors. Fractures at both Almen intensities exhibit radial characteristics, with the arrow indicating the crack that initiates below the surface. Shot peening is a well-known surface treatment to prevent crack initiation and propagation in components subjected to fatigue loading, similar to the study by V. Fridrici^[33]^. The crack initiation region appears relatively smooth and flat due to high cyclic loading. Compared to the high Almen intensity, the surface of the fatigue source area is relatively rough, and the crack initiation point is closer to the edge of the low Almen intensity specimens. Figure 4d, e show the micro-morphology of the final fracture area under different Almen intensities. Both regions exhibit a characteristic distribution of dimples, which are similar in size. These indicators characterize ductile fracture.

### Plastic strain range

**Fig. 5:**
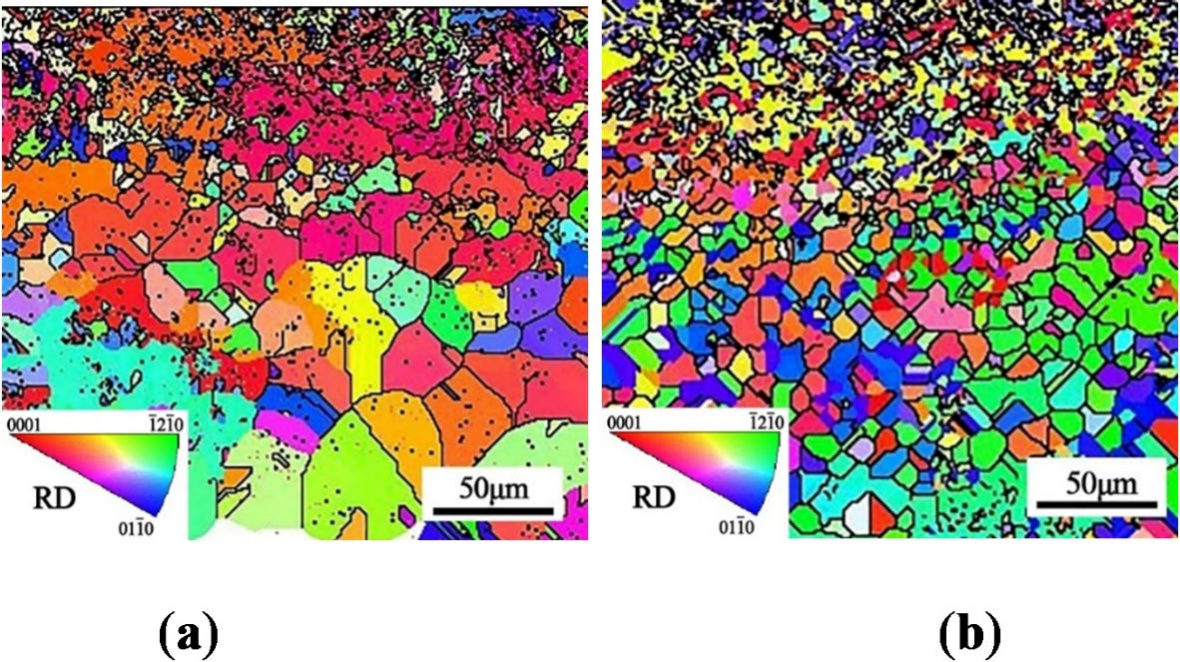
EBSD inverse pole figure (IPF) maps with different Almen intensities: (**a**) 0.15 mmA; (**b**) 0.25 mmA.

**Fig. 6:**
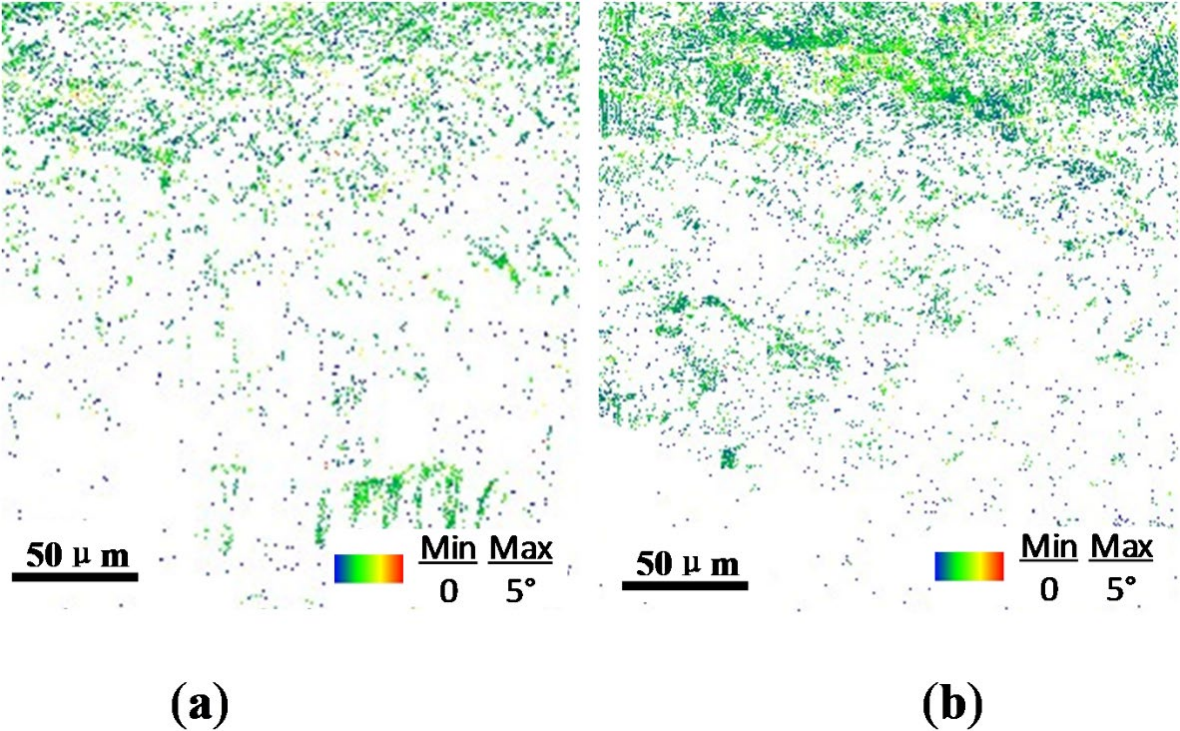
EBSD kernel average misorientation (KAM) maps with different Almen intensities: (**a**) 0.15 mmA; (**b**) 0.25 mmA.

The inverse pole figure (IPF) maps of the cross-section of TC17 alloy are presented in Fig. 5, where different colors reflect the orientation of the grains in the specimen cross-section. Within the characterization range of 200 μm, the grains significantly rotate near the surface layer, indicating stronger plastic deformation after the USP treatment. The grain size induced by the USP treatment gradually decreases with increased layer depth, and the subsurface layer shows a gradient distribution of grain size from the surface layer to the matrix. The Kernel-Average-Misorientation (KAM) distribution diagram of the cross-section of TC17 alloy is shown in Fig. 6. Many researchers have emphasized using Electron Backscatter Diffraction (EBSD) to measure strain in deformed polycrystalline materials^34–36^. There are various ways to present measurements from EBSD datasets, and EBSD patterns have been used to quantify plastic deformation in specimens^34^. The KAM maps qualitatively reflect the degree of homogenization of plastic deformation, where a higher value indicates a higher degree of plastic deformation or a higher defect density. Due to the high degree of grain refinement and high grain boundary density induced by USP treatment on the surface layer, the local area data is difficult to index and appears white, as observed in Rochlus’s study^37^. The plastic strain distribution caused by Almen intensities of 0.15 mmA and 0.25 mmA is evident at approximately 70 μm and 100 μm below the surface, respectively. Similar to the study by Messé O^38^, surface strengthening induced an increase in the strained layer to a depth of approximately 60 μm. The distribution range of the strained layer is similar to the depth of the grain refinement layer, and this region accounts for a small proportion of the entire depth region. The color distribution of the deformation area shows that the plastic deformation caused by USP treatment has a uniform distribution in the lateral direction, which meets the uniform distribution of plastic strain.

**Fig. 7:**
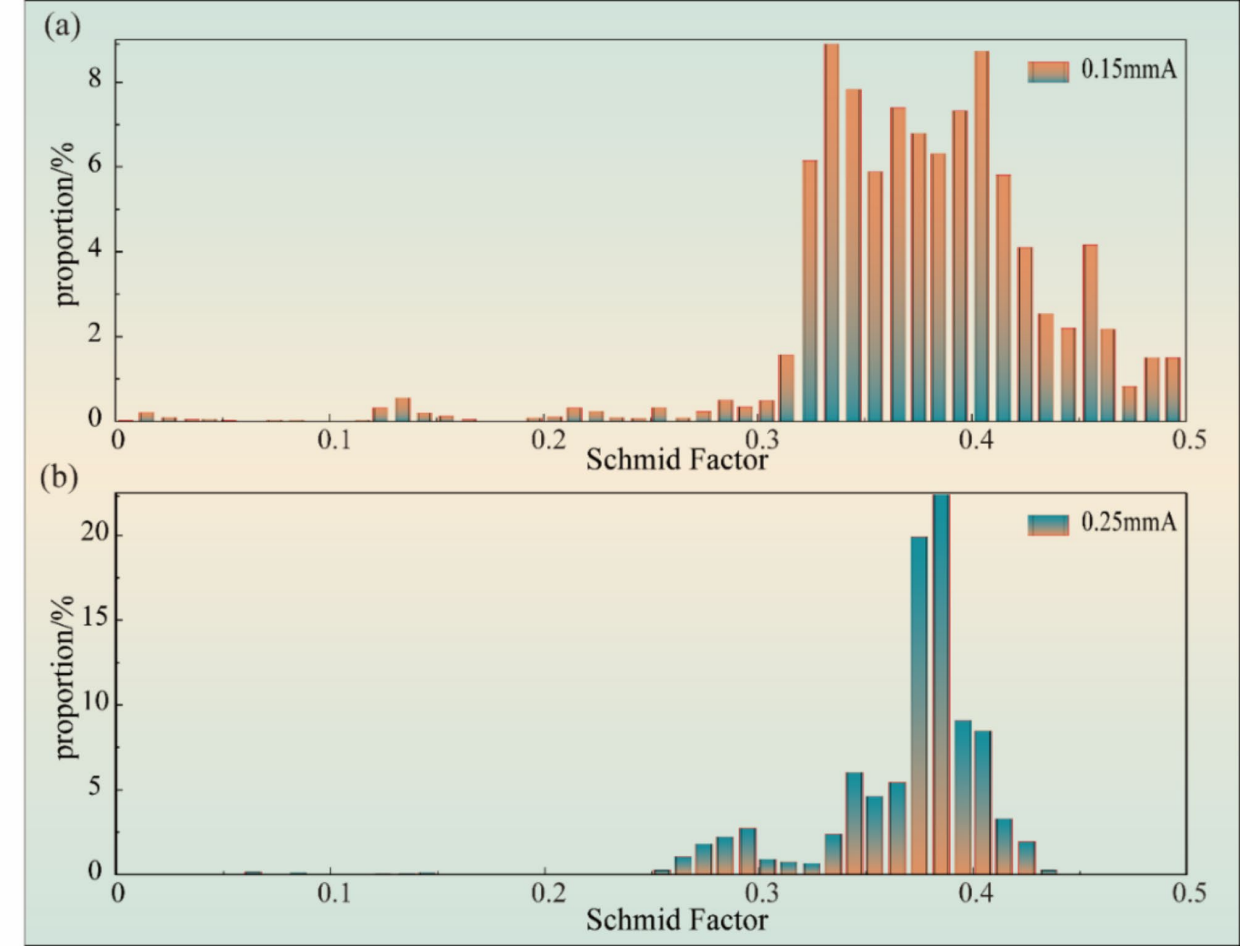
Distribution of Schmid factor of TC17 alloy with different Almen intensities: (**a**) 0.15 mmA; (**b**) 0.25 mmA.

The distribution of the Schmid factor (SF) under different Almen intensities is presented in Fig. 7. The close-packed slip surface of the α phase is analyzed, and the SF distribution of the basal plane {0001}, cylinder plane {1-100}, and the cone plane {1-101} in the < 11–20 > direction under the range of (0, 0.5) is compared. It is concluded that the cone plane {1-101} is the close-packed slip surface of the α phase of the TC17 alloy. The SF distribution states under different Almen intensities are analyzed based on the crystal plane family. The results show that for the 0.15 mmA specimen, the proportion of SF = 0.35 is the largest. When the Almen intensity is 0.25 mmA, the proportion of SF = 0.37 is the largest, with a decrease in the number of soft-oriented grains. The size of SF characterizes the difficulty of plastic deformation, where a large SF value indicates that the slip system is easy to start and prone to plastic deformation, while a small SF value indicates that the slip system is difficult to start and plastic deformation is difficult. The SF distribution decreases compared to the 0.15 mmA specimen and is characterized as a hard orientation state for the 0.25 mmA specimens. If the SF partially reaches 0.5 when the Almen intensity is 0.15 mmA, the distribution is classified as a soft orientation state. The plastic layer introduced under the aforementioned Almen intensity is relatively small, the degree of work hardening is low, and slip deformation is more likely to occur.

**Fig. 8:**
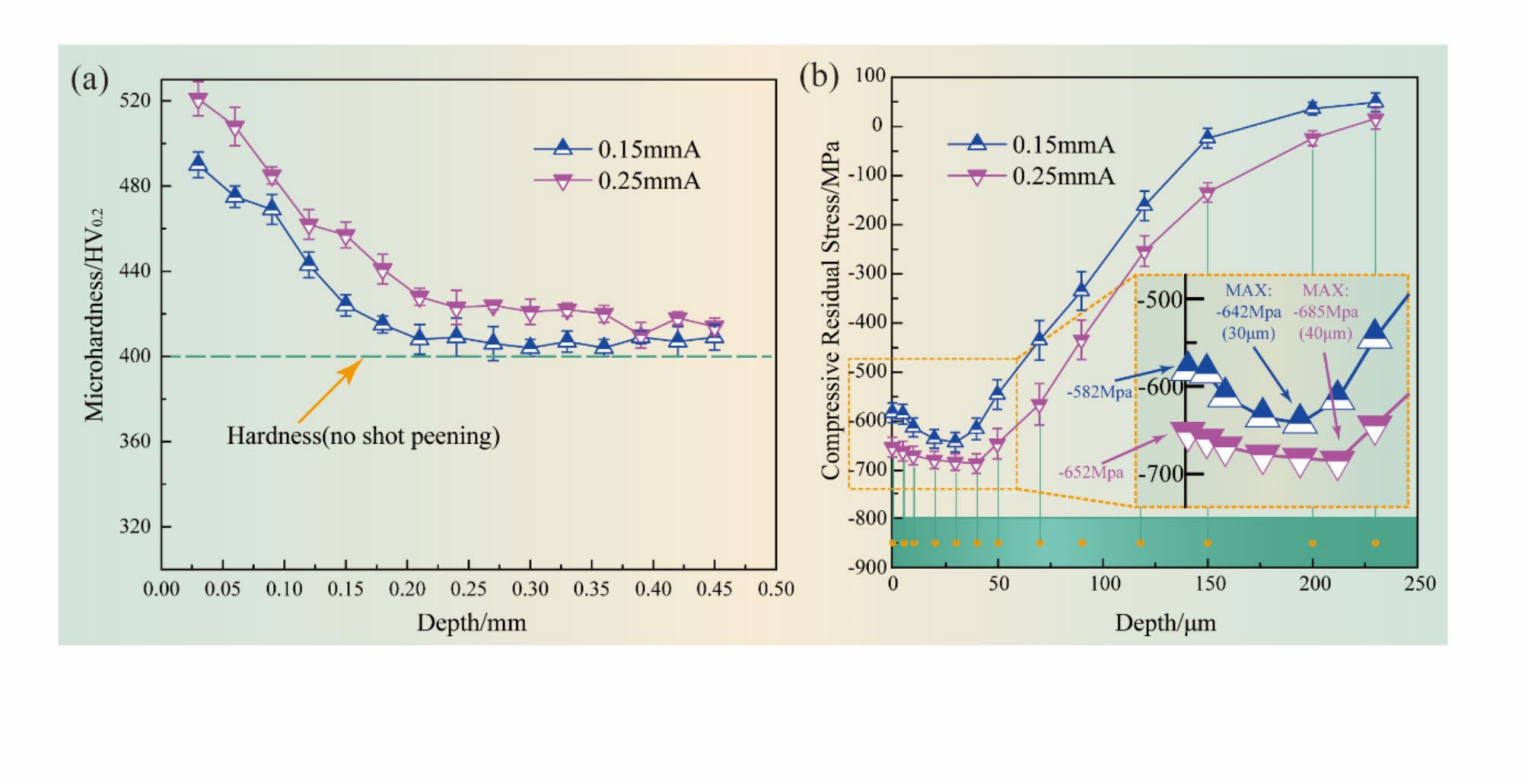
Depth distribution of microhardness and residual stress layer of specimens with different Almen intensities: (**a**) microhardness; (**b**) residual stress.

Figure 8a illustrates the in-depth microhardness distributions for the TC17 alloy subjected to various surface treatments. The in-depth microhardness distributions follow a similar pattern after different processes, gradually decreasing with increasing depth and eventually reaching and remaining at the level of the matrix microhardness (400HV_0.2_). A pronounced decrease in microhardness is observed within the depth of 0.20 μm. All the surface microhardness values are improved by the different USP processes. As a result of the induced plastic deformation, a work-hardened layer gradually forms in the subsurface region. The work hardening layer is formed by the combination of the properties of the material itself and the plastic deformation of the surface during the treatment process. In comparison with matrix microhardness, surface microhardness values induced by the 0.25mmA and 0.15mmA are notably improved by 30.3% and 22.5%, respectively. This observation indicates that microhardness can be effectively enhanced by increasing the Almen intensities. The distribution curve of residual stress along the layer depth for different Almen intensities is presented in Fig. 8b. The results show that when the Almen intensity is increased, the depth of the residual compressive stress layer significantly increases. The maximum compressive residual stress and depth variation value are relatively small. For different Almen intensities, the depth of the compressive residual stress layer extends to the subsurface by approximately 100 μm due to the grain refinement layer. Based on the analysis of Fig. 4, it can be concluded that the inhibition of crack initiation by the compressive residual stress layer increases under low loads and high cycles, as opposed to high load conditions. Following the USP treatment, the compressive residual stress affects the crack initiation point, extending in the depth direction. This inhibits the initiation of cracks from the surface and avoids the fracture of titanium alloy test rods in the early stage of fatigue^39^.

## Conclusion

This paper investigates the impact of USP on the surface roughness, microstructure, and fatigue behavior of TC17 alloy. Based on the experimental results and analysis, the following conclusions can be drawn:

The subsurface region of the specimen treated by USP exhibited a gradual distribution of grain sizes, with a decrease in grain size towards the surface and an increase towards the depth. An increase in Almen intensity resulted in a more extensive distribution of the grain gradient along the depth direction, and the grain deformation layer induced by USP treatment was observed to be more widely distributed.

The inhibitory effect of the compressive residual stress layer on crack initiation increased for low loads and high cycle fatigue. The depth of the fatigue crack initiation point from the surface was observed to increase with increasing Almen intensity. Moreover, the increase in the depth of the compressive residual stress layer induced by USP treatment was found to counteract the early fatigue failure caused by higher roughness.

The concentration distribution range of the Schmid factor exhibited a decreasing trend, and the work hardening induced by USP treatment reduced the propensity for slip deformation under both Almen intensities.

## Data Availability

The authors declare that the data supporting the findings of this study are available within the paper and Should any raw data files be needed in another format they are available from the corresponding author upon reasonable request.

## References

[1] Chen, Y., Yang, X. & Wei, F. Development trend of high bypass ratio turbofans design technology. *AcAAS* (2017).

[2] Chamis., C. Vibration characteristics of composite fan blades and comparison with measured data. *J. Aircr.* **14**, 644–647. 10.2514/3.58835 (1976).

[3] Cowles, B. A. High cycle fatigue in aircraft gas turbines—an industry perspective. *Int. J. Fatigue*. **80**, 147–163. 10.1016/S0142-1123(98)91116-1 (1996).

[4] Altenberger, I. Alternative mechanical surface treatments: microstructures, residual stresses & fatigue behavior. *Shot Peening* 419–434. 10.1002/3527606580.ch54 (2003).

[5] Luo, J. & Bowen, P. Effects of temperature and shot peening on S-N behavior of a PM Ni-base superalloy UDIMET 720. *Metall. Mater. Trans. A*. **35**, 1007–1016. 10.1007/s11661-004-1004-9 (2004).

[6] Gibson, G. J., Perkins, K. M., Gray, S. & Leggett, A. J. Influence of shot peening on high-temperature corrosion and corrosion-fatigue of nickel based superalloy 720Li. *Mater. High. Temp.* **33**, 225–233. 10.1080/09603409.2016.1161945 (2016).

[7] Nalla, R. K. et al. On the influence of mechanical surface treatments—deep rolling and laser shock peening—on the fatigue behavior of Ti–6Al–4V at ambient and elevated temperatures. *Mater. ScienceEngineering: A*. **355**, 216–230. 10.1016/S0921-5093(03)00069-8 (2003).

[8] Liu, Y., Mcgrory, B. & Mcgrory, C. Nanocrystallisation on the surface of superalloy In718 component with a commercial shot peening process. *International Conference of Shot Peening* (2018).

[9] Tsuji, N., Tanaka, S. & Takasugi, T. Effects of combined plasma-carburizing and shot-peening on fatigue and wear properties of Ti–6Al–4V alloy. *Surf. Coat. Technol.* **203**, 1400–1405. 10.1016/j.surfcoat.2008.11.013 (2009).

[10] Prevey, P. S., Ravindranath, R. A., Shepard, M. & Gabb, T. Case studies of fatigue life improvement using low plasticity burnishing in gas turbine Engine Applications. *ASME*10.1115/1.1807414 (2003).

[11] Evans, A., Kim, S. B. & Shackleton, J. Relaxation of residual stress in shot peened Udimet 720Li under high temperature isothermal fatigue. *Int. J. Fatigue*. **27**, 1530–1534. 10.1016/j.ijfatigue.2005.07.027 (2005).

[12] J Foss, B. et al. Analysis of shot-peening and residual stress relaxation in the nickel-based superalloy RR1000. *AcMat* **61**, 2548–2559. 10.1016/j.actamat.2013.01.031 (2013).

[13] Dekhtyar, A. et al. Enhanced fatigue behavior of powder metallurgy Ti–6Al–4V alloy by applying ultrasonic impact treatment. *Mater. Sci. Engineering: A*. **641**, 348–359. 10.1016/j.msea.2015.06.072 (2015).

[14] Mordyuk, B., Dekhtyar, A., Savvakin, D. & Khripta, N. Tailoring porosity and microstructure of alpha-titanium by combining powder metallurgy and ultrasonic impact treatment to control elastic and fatigue properties. *J. Mater. Eng. Perform.* **31**, 5668–5678. 10.1007/s11665-022-06633-7 (2022).

[15] Watanabe, Y. et al. Effect of ultrasonic shot peening on fatigue strength of high strength steel. *Shot Peening* 305–310. 10.1002/3527606580.ch38 (2003).

[16] Goulmy, J. P. et al. A calibration procedure for the assessment of work hardening part II: application to shot peened IN718 parts. *Mater. Charact.* **175**10.1016/j.matchar.2021.111068 (2021).

[17] Stoll, I., Helm, D., Polanetzki, H. & Wagner, L. Ultrasonic shot peening (USP) on Ti-6Al-4 V and Ti-6Al-2Sn-4Zr-6Mo aero engine components enginelease aero. *The 11th ICSP South Bend* (2011).

[18] Mapelli, C. et al. Survey about effects of shot peening conditions on fatigue performances of Ti–6Al–4V mechanical specimens featured by different cross-section geometries. *Mater. Sci. Technol.* **28**, 543–548. 10.1179/1743284711Y.0000000096 (2012).

[19] Zhang, Q., Duan, B., Zhang, Z., Wang, J. & Si, C. Effect of ultrasonic shot peening on microstructure evolution and corrosion resistance of selective laser melted Ti–6Al–4V alloy. *J. Mater. Res. Technol.* **11**, 1090–1099. 10.1016/j.jmrt.2021.01.091 (2021).

[20] Tan, M., Cai, J., Qu, J., Xie, X. & Lv, S. Effect of surface strengthening by ultrasonic shot peening on TC17 alloy. *J. Surf. Topography: Metrol. Prop.* **12**, 015017. 10.1088/2051-672X/ad2cb4 (2024).

[21] Wang, Y. Numerical simulation analysis of residual stress in ultrasonic shot peening of TC4 titanium alloy. *Aeroengine* **45**, 58–64 (2019). 10.13477/j.cnki.aeroengine.2019.03.010

[22] Xu, Q., Cao, Y., Cai, J., Yu, J. & Si, C. The influence of ultrasonic shot peening on the surface roughness, microstructure, and mechanical properties of TC2 thin-sheet. *J. Mater. Res. Technol.* **15**, 384–393. 10.1016/j.jmrt.2021.08.029 (2021).

[23] Cai, J., Cherutich, K. C., Li, W., Shi, J. D. & Lin, S. Surface Roughness Numerical and Test evaluation of FGH97 powder superalloy by Ultrasonic Shot Peening. *Surf. Technol.* **50**, 250–257. 10.16490/j.cnki.issn.1001-3660.2021.06.028 (2021).

[24] Rodopoulos, C., Kermanidis, A. T., Statnikov, E., Vityazev, V. & Korolkov, O. The effect of surface engineering treatments on the fatigue behavior of 2024-T351 aluminum alloy. *J. Mater. Eng. Perform.* **16**, 30–34. 10.1007/s11665-006-9004-0 (2007).

[25] Gao, Y. Improvement of fatigue property in 7050–T7451 aluminum alloy by laser peening and shot peening. *Mater. Sci. Eng. A*. **528**, 3823–3828. 10.1016/j.msea.2011.01.077 (2011).

[26] Zhu, L. et al. Influence of process parameters of ultrasonic shot peening on surface roughness and hydrophilicity of pure titanium. *J. Aircr.* **317**, 38–53. 10.1016/j.surfcoat.2017.03.044 (2017).

[27] Zhang, Q. et al. Residual stress and microhardness evolution induced by conventional and ultrasonic shot peening. *Mater. Sci. Technol.* **38**, 38. 10.1080/02670836.2022.2045551 (2022).

[28] Mordyuk, B., Prokopenko, G., Milman, Y. V., Iefimov, M. & Sameljuk, A. Enhanced fatigue durability of Al–6 mg alloy by applying ultrasonic impact peening: effects of surface hardening and reinforcement with AlCuFe quasicrystalline particles. *Mater. Sci. Eng. A*. **563**, 138–146. 10.1016/j.msea.2012.11.061 (2013).

[29] Zhang, Q. et al. Microstructure change and corrosion resistance of selective laser melted Ti-6Al-4V alloy subjected to pneumatic shot peening and ultrasonic shot peening. *Surf. Topogr*. **10**10.1088/2051-672X/ac4c83 (2022).

[30] Kumar, S. et al. Effect of surface nanostructure on tensile behavior of superalloy IN718. *Mater. Des.* **62**, 76–82. 10.1016/j.matdes.2014.04.084 (2014).

[31] Pandey, V., Singh, J., Chattopadhyay, K., Srinivas, N. S. & Singh, V. Influence of ultrasonic shot peening on corrosion behavior of 7075 aluminum alloy. *J. Alloys Compd.* **723**, 826–840. 10.1016/j.jallcom.2017.06.310 (2017).

[32] Wu, D., Yao, C. & Zhang, D. Surface characterization and fatigue evaluation in GH4169 superalloy: comparing results after finish turning; shot peening and surface polishing treatments. *Int. J. Fatigue*. **113**, 222–235. 10.1016/j.ijfatigue.2018.04.009 (2018).

[33] Fridrici, V., Fouvry, S. & Kapsa, P. Effect of shot peening on the fretting wear of Ti–6Al–4V. *Wear*10.1016/S0043-1648(01)00671-8 (2001).

[34] Yoda, R., Yokomaku, T. & Tsuji, N. Plastic deformation and creep damage evaluations of type 316 austenitic stainless steels by EBSD. *Mater. Charact.* **61**, 913–922. 10.1016/j.matchar.2010.05.006 (2010).

[35] Kamaya, M., Wilkinson, A. J. & Titchmarsh, J. M. Quantification of plastic strain of stainless steel and nickel alloy by electron backscatter diffraction. *AcMat*10.1016/j.actamat.2005.08.046 (2006).

[36] Kamaya, M., Wilkinson, A. J. & Titchmarsh, J. M. Measurement of plastic strain of polycrystalline material by electron backscatter diffraction. *Nucl. Eng. Des.* **235**, 713–725. 10.1016/j.nucengdes.2004.11.006 (2005).

[37] Rochlus, K., Slawik, S. & Muecklich, F. Influences of the Edge Area Preparation suitable for EBSD of Ferrous materials of different degrees of hardness. *Pract. Metallogr*. **53**, 393–407. 10.3139/147.110405 (2016).

[38] Messé, O. M. D. M., Stekovic, S., Hardy, M. C. & Rae, C. M. F. Characterization of Plastic Deformation Induced by Shot-Peening in a Ni-Base superalloy. *JOM* **66**, 2502–2515. 10.1007/s11837-014-1184-8 (2014).

[39] Tan, L., Zhang, D., Yao, C., Wu, D. & Zhang, J. Evolution and empirical modeling of compressive residual stress profile after milling, polishing and shot peening for TC17 alloy. *Metall. Mater. Trans. A*. **26**, 155–165. 10.1016/j.jmapro.2017.02.002 (2017).

